# Indoor Intruder Tracking Using Visible Light Communications

**DOI:** 10.3390/s19204578

**Published:** 2019-10-21

**Authors:** Farah M. Alsalami, Zahir Ahmad, Stanislav Zvanovec, Paul Anthony Haigh, Olivier C. L. Haas, Sujan Rajbhandari

**Affiliations:** 1 Research Institute for Future Transport and Cities, Coventry University , Coventry CV1 5FB, UK; alsallaf@uni.coventry.ac.uk (F.M.A.);; 2 Faculty of Engineering, Environment & Computing, Coventry University, Coventry CV1 5FB, UK; ab7175@coventry.ac.uk; 3 Department of Electromagnetic Field, Faculty of Electrical Engineering, Czech Technical University in Prague, Technicka , Prague 16627, Czech Republic; xzvanove@fel.cvut.cz; 4 Intelligent Sensing and Communications Group, Newcastle University, Newcastle-upon-Tyne NE1 7RU, UK; Paul.Haigh@newcastle.ac.uk

**Keywords:** indoor VLC channel, Kalman filter, minimax filter, state estimation, visible light communication, intruder tracking

## Abstract

This paper proposes a comprehensive study of indoor intruder tracking using visible light communication (VLC). A realistic indoor VLC channel was developed, taking into consideration reflections, shadowing, and ambient noise. The intruder was considered smart and aiming to escape tracking. This was modelled by adding noise and disturbance to the intruder’s trajectory. We propose to extend the application of minimax filtering from state estimation in the radio frequency (RF) domain to intruder tracking using VLC. The performance of the proposed method was examined and compared with Kalman filter for both VLC and RF. The simulation results showed that the minimax filter provided marginally better tracking and was more robust to the adversary behavior of the intruder than Kalman filter, with less than 0.5 cm estimation error. In addition, minimax was significantly better than Kalman filter for RF tracking applications.

## 1. Introduction

Visible light communication (VLC) can be a competent technology for both energy-efficient illumination and reliable data communication [1,2]. In addition to broadband communication, this combined lighting and communication system has low power consumption and immunity to interference from radio frequencies. Therefore, VLC systems have gained increasing interest in indoor and outdoor applications such as positioning, localisation and tracking [3,4].

Target tracking for security and real-time surveillance application has crucial importance, particularly if the target is intelligent. In such applications, the target has adversarial behaviors, such as adding arbitrary disturbance to escape the tracking. Therefore, there is an essential requirement for an effective real-time technique to track this type of intruder for security and safety applications.

VLC-based indoor positioning techniques and tracking systems are mostly based on the received signal strength (RSS) [5,6], time of arrival (TOA) [7,8,9] and angle of arrival (AOA) [10]. The study in [11] proposed an intelligent photodiode (PD) system that utilises light-emitting diode (LED) coverage to identify the location and track the position. Additionally, it has the ability to adjust the horizontal and vertical field of view (FOV) of the signal reception to reduce the interference. A maximum accuracy of 0.03 m was achieved for a user with a speed of 0.3 m.s${}^{-1}$ in a room with dimensions 7×7×3 m${}^{3}$. Authors in [12] proposed a receiver system with three tilted PDs facing in different directions and an accelerometer to calculate the orientation and coordinates of the receiver. The study reported an accuracy of 0.06 m when the receiver was moving at an average speed of 1.3 m.s${}^{-1}$ in a room with dimensions 5×4×3 m${}^{3}$. The work in [13] utilises a fish-eye lens camera in conjunction with the Levenberg–Marquardt algorithm to estimate the receiver position with an accuracy better than 0.01 m. The authors in [14] carried out a comparative study of a Kalman filter and sequential importance sampling particle filters for positioning and tracking by measuring the RSS. The study concluded that the particle filter outperformed the Kalman filter at the expense of additional computational complexity. In [15], a spatial diversity VLC transmitter with recursive state estimation using Kalman filter demonstrated that an accuracy better than 0.05 m was achievable in an empty room with dimensions 6×6×4 m${}^{3}$. The study in [16] also uses Kalman filter for indoor VLC tracking to mitigate the optical link blockage and the blur effect due to high-speed movement when the image sensor is used as a receiver. The study exploits optical flow to detect the target position and a Bayesian algorithm to predict the next step in the target movement. The output of the detector and the predictor are fused as an input to the Kalman filter in order to calculate the target trajectory. An accuracy better than 0.01 m was achieved for a target speed of up to 13.3 m.s${}^{-1}$ in an experimental platform of size 1.9×1×1.9 m${}^{3}$.

These studies have shown the feasibility of accurately estimating the location of an ordinary target using VLC, but only for the cases where the target cooperates and calculates its own position. However, none of these studies have addressed indoor intruder tracking for real-time surveillance and building security purposes using a realistic indoor VLC channel model. In the latter case, the tracking needs to be done without the knowledge of the intruder and without any active device fitted to the intruder. Further, a smart intruder has a random and adversary mobility pattern requiring the effect of multipath propagation and shadowing to be taken into account. These assumptions invalidate the simplified assumptions of the line of sight (LOS) link [17,18] and require a wide-FOV receiver. The minimax filter was proposed to track adversary targets within the radio frequency (RF) domain [19,20,21,22,23] and was reported to outperform Kalman filter when the target was an adversary with an unpredictable noise model. Particularly, the minimax filter has bounded estimation error values in contrast to the Kalman filter [19]. However, there is very limited work on the use of the minimax filter for VLC [24].

Therefore, the contributions of this paper are summarised as follows:

- A VLC-based system for adversary target tracking inside buildings. A minimax filter is proposed to overcome possible failures of classical tracking methods in the case of an intelligent adversarial target. To the best knowledge of the authors, this is the first comprehensive study of adversary target tracking inside buildings using a VLC system.
- The simulation is based on a realistic channel model that takes multipath propagation, mobility of objects and shadowing into consideration.
- We examined the performance of the minimax filter against the Kalman filter, which is used as a benchmark, for the VLC-based tracking system, in terms of tracking accuracy and calculation complexity performance. The results showed that the minimax filter provided marginal tracking accuracy compared to the Kalman filter. However, both filters had a similar level of calculation complexity.
- This is the first study to compare the performance of both Kalman and minimax filters for RF- and VLC-based intruder tracking, taking into consideration the inherent differences in noise and position measurement accuracy. VLC is expected to provide more accurate position measurements in comparison to RF. Hence, the conclusion drawn in the RF domain is not necessarily applicable to VLC. For example, in RF, minimax is necessary to track the adversary target as Kalman filter is inadequate [2]. However, this study shows that the Kalman filter was adequate for intruder tracking with the VLC system.

The rest of the paper is organised as follows: Section 2 presents the system including the complete derivation of the mathematical model for the channel and the target movement. In Section 3, the design of filters is presented. Section 4 evaluates the performance of the proposed system through simulation. Finally, concluding remarks are presented in Section 5.

## 2. System Description

**Figure 1:**
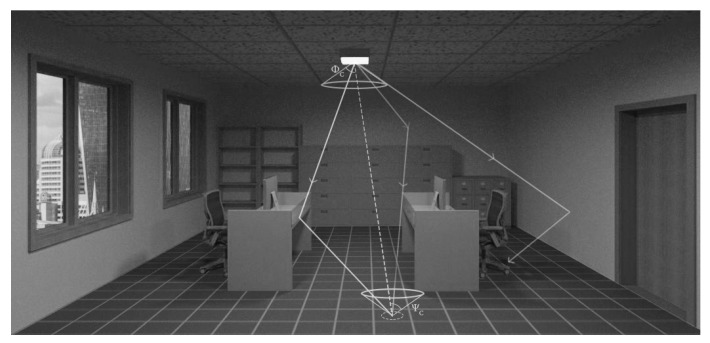
A schematic of the proposed indoor intruder tracking using visible light communication (VLC) showing three possible ray paths: line of sight (LOS), reflection from walls and furniture and blocked rays.

Figure 1 shows a schematic of the VLC system under study. The system consists of a typical indoor room environment with a LED-based illumination source in the ceiling pointing downwards as a transmitter. Receivers are placed on the floor, pointing upwards. The figure shows one receiver as an example. For simplicity and cost-effectiveness, we assumed receivers with a PIN PD and field-effect transistor (FET)-based transimpedance amplifier.

When the intruder enters the detection zone, the nearest PD detects the position of the target at any instant. Then, this PD sends the position coordinates to a central processing node, which estimates the target trajectory. The number of PDs depends on the detection area and the stride length of the expected target (e.g., a robot or human).

The goal of this study was to evaluate the performance of tracking algorithms in estimating the trajectory of an intruder using the VLC system. Without applying the tracking algorithm, the trajectory of the intruder is unknown, and the central processing node stores discrete position information. The detailed trajectory model and tracking algorithm are described below. Section 2.1 describes the indoor channel model and its impact on the system. Section 2.2 presents the adversary target movement model.

### 2.1. Indoor Channel Model

The accuracy of the target positioning is influenced by the signal-to-noise ratio (SNR) at the receiver. Two factors affect the SNR at the receiver: (a) the channel DC gain that depends on the surrounding environment (e.g., room dimension, shadowing and reflection) and (b) noise sources.

The proposed room scenario (Figure 1) takes into account the effect of the ambient light coming from the two windows, the effect of reflection from the walls and the furniture and the effect of rays blockage by objects and obstacles.

The channel impulse response (CIR) is used to characterise the channel condition and identify the relation between the transmitted optical signal power and the received optical signal. The CIR in the VLC system depends on the LOS $h_{LOS}$, multipath $h_{ref}$ and shadowing $h_{shd}$ components as given by:

(1) $$\begin{array}{c} \begin{array}{c} h\left(t\right)=h_{LOS}\left(t\right)+h_{ref}\left(t\right)+h_{shd}\left(t\right), \end{array} \end{array}$$

where *t* is the time.

The DC gain for LOS $h_{LOS}$ propagation for a LED with a Lambertian radiation pattern is given by [25]:

(2) $$\begin{array}{c} \begin{array}{c} h_{LOS}\left(t\right)=\frac{A_{r}\left(m+1\right)}{2\pi d^{2}}\cos^{m}\left(\phi \right)T_{s}\left(\theta \right)g\left(\theta \right)\cos\left(\theta \right)\delta \left(t-\frac{d}{c}\right), \end{array} \end{array}$$

where $A_{r}$ is the PD active area, $T_{s}\left(\theta \right)$ and g(θ) are optical filter and concentrator gain, respectively, *d* is the distance between transmitter and receiver, ϕ is the irradiance angle and θ is the incidence angle. *m* is the order of emission which depends on the semi-angle at a half illuminance $\Phi_{1/2}$ of a LED and is given by $m=\frac{ln(2)}{ln(cos\Phi_{1/2})}$. The expression $\delta (t-\frac{d}{c})$ is the propagation delay of the signal and *c* is the speed of light. Assuming Lambertian reflection from walls and furniture, the multipath impulse response component after the $r^{\mathrm{th}}$ reflection is given by [25]:

(3) $$\begin{array}{c} \begin{array}{c} h_{ref}\left(t\right)=\frac{\left(m+1\right)}{2\pi }\sum_{j=1}^{R_{r}}A_{j}\rho_{j}\cos^{m}\left(\phi_{j}\right)\frac{\cos(\theta )}{d_{j}^{2}}rect\left(\frac{2\theta }{\pi }\right)h_{ref}^{r-1}\left(t-\frac{d_{j}}{c}\right), \end{array} \end{array}$$

where $R_{r}$ is the number of reflectors, $j\epsilon [1,R_{r}]$ is the reflector index, $\rho_{j}$ is the reflection coefficient of the surfaces including walls of the $j^{\mathrm{th}}$ reflector, $A_{j}$ is the area of the $j^{\mathrm{th}}$ reflector, rect() is a rectangular function which indicates that the PD detected light within an incidence angle less than π/2 [25] and $h_{ref}^{r-1}\left(t-\frac{d_{j}}{c}\right)$ is the r−1 order impulse response.

Likewise, the shadowing component can be accounted for in (3) by including a blocking probability $O_{i}$ as in [18]:

(4) $$\begin{array}{c} \begin{array}{c} h_{shd}\left(t\right)=\frac{\left(m+1\right)}{2\pi }\sum_{i=1}^{N_{t}}A_{i}\rho_{i}\cos^{m}\left(\phi_{i}\right)\frac{\cos(\theta )}{d_{i}^{2}}rect\left(\frac{2\theta }{\pi }\right)h_{shd}^{r-1}\left(t-\frac{d_{i}}{c}\right)O_{i}. \end{array} \end{array}$$

The blocking probability $O_{i}$ of the blocking object *i* has a Poisson distribution for a random number of obstacles $N_{t}$ of intensity ϵ is given by [18]:

(5) $$\begin{array}{c} \begin{array}{c} O_{i}=\exp\left[-\epsilon t\left(\int_{0}^{c_{x}}\int_{0}^{c_{y}}\left(\int_{2d(c_{x},c_{y})}^{\infty }\int_{s(c_{x},c_{y})}^{\infty }g\left(w_{o},h_{o}\right)dw_{o}\;dh_{o}\right)\;f\left(c_{x},c_{y}\right)\;dc_{x}\;dc_{y}\right)\right], \end{array} \end{array}$$

where $c_{x}$ and $c_{y}$ denote the position coordinates of the obstacle *i*, $w_{o}$ and $h_{o}$ are the dimensions of the obstacle *i*, $d(c_{x},c_{y})$ and $s(c_{x},c_{y})$ are the coordinates of the ray, $g(w_{o},h_{o})$ and $f(c_{x},c_{y})$ are the joint probability density functions of the obstacle size and location, respectively. The shot noise from the natural source (e.g., sunlight) and/or artificial illumination sources and thermal noise affect the performance of the VLC. The total noise variance $\sigma_{total}^{2}$ is given by:

(6) $$\begin{array}{c} \begin{array}{c} \sigma_{total}^{2}=\sigma_{shot}^{2}+\sigma_{th}^{2}, \end{array} \end{array}$$

where $\sigma_{shot}^{2}$ and $\sigma_{th}^{2}$ are the shot and thermal noise variances, respectively. The ambient light from the windows and other natural or artificial light sources induces a shot noise, which is given by [25]:

(7) $$\begin{array}{c} \begin{array}{c} \sigma_{shot}^{2}=2qP_{R}\gamma B_{w}+2qI_{bg}I_{2}B_{w}, \end{array} \end{array}$$

where $P_{R}$ is the received power, γ is the PD responsivity, *q* is the electron charge, $I_{2}$ is the charge noise-bandwidth factor, $I_{bg}$ is background current and $B_{w}$ is the noise bandwidth.

The thermal noise is given by [25]:

(8) $$\begin{array}{c} \begin{array}{c} \sigma_{th}^{2}=\frac{8\pi K_{k}T_{k}C_{pd}A_{r}I_{2}B_{w}^{2}}{G_{\nu }}+\frac{16\pi^{2}K_{k}T_{k}\Gamma C_{pd}^{2}A_{r}^{2}I_{3}B_{w}^{3}}{g_{m}}, \end{array} \end{array}$$

where $K_{k}$ is the Boltzmann’s constant, $T_{k}$ is the absolute temperature, $C_{pd}$ is the fixed capacitance of PD per unit area, $I_{2}$ and $I_{3}$ are the noise bandwidth factors, $G_{\nu }$ is the open-loop voltage gain, Γ is the FET channel noise factor and $g_{m}$ is the FET transconductance.

Having presented the indoor channel model describing the interaction between emitters, detectors and their environment, the next subsection focuses on the intruder motion model.

### 2.2. Target Movement Model

The states of the target motion were modelled as a discrete linear time-invariant system [21]:

(9) $$x_{k+1}={\mathrm{Ax}}_{k}+{\mathrm{Bw}}_{k},$$

where *k* is the discrete-time index, $x_{k}$ is the matrix of the system state and $w_{k}$ is the matrix of target acceleration. A is the state transition matrix that describes the relation of the system state at time *k* to the next step at time k+1, that is, after a specific duration Δt [19,20]:

$$A=\left[\begin{array}{cccc} 1 & 0 & \Delta t & 0\\ 0 & 1 & 0 & \Delta t\\ 0 & 0 & 1 & 0\\ 0 & 0 & 0 & 1 \end{array}\right].$$

B is a matrix that relates target acceleration to the actual state value $x_{k}$ and it is given by [19,20]:

$$B=\left[\begin{array}{cc} 0.5\Delta t^{2} & 0\\ 0 & 0.5\Delta t^{2}\\ \Delta t & 0\\ 0 & \Delta t \end{array}\right].$$

The target can have constant velocity (zero acceleration) or constant acceleration. However, the realistic case is to have a random acceleration. In this case, acceleration can be modelled as a Gaussian random variable $w_{k}$ which is modelled by a 2×1 matrix of a Gaussian random variable with zero mean and covariance matrix Q>0, that is:

$$w_{k}=\left[\begin{array}{c} A_{x}\left(t\right)\\ A_{y}\left(t\right) \end{array}\right].$$

Further, we assumed that the target is adversary intelligent and can escape from the tracking by adding additional disturbance $\Delta_{k}$. Then, the motion model becomes [19,20]:

(10) $$x_{k+1}={\mathrm{Ax}}_{k}+{\mathrm{Bw}}_{k}+\Delta_{k}.$$

$\Delta_{k}$ parameterises the adversary behavior of the intruder, which is modelled by the following matrix:

(11) $$\Delta_{k}=L\left(G\left(x_{k}-\hat{x_{k}}\right)+n_{k}\right),$$

where $n_{k}$ is the Gaussian noise with zero mean and a covariance matrix S>0, L is the adversary gain that can be added by the intruder, $\hat{x_{k}}$ is the estimated state, $x_{k}-\hat{x_{k}}$ is the estimation error and G is the weighting matrix that indicates the adversary behavior. The intruder aims to maximise the estimation error by G. If L or G equal to zero, then the intruder becomes an ordinary target with a conventional motion model [20].

According to the scenario, illustrated in Figure 1, the states of the target are measured by PDs placed on the floor. When the target enters the detection area of any PD, this PD sends its position coordinates $y_{k}$ to the central processing node. The state measurement is corrupted by additive noise generated by external and internal sources. Accordingly, the measurements equation is given by:

(12) $$y_{k}={\mathrm{Cx}}_{k}+v_{k},$$

where C is the matrix that relates the measurement value $y_{k}$ to the actual state value $x_{k}$, $v_{k}$ is the Gaussian noise matrix with zero mean and a covariance matrix R>0.

If the measurements in the *x* and *y* dimensions are independent, then the covariance matrix R of the measurement noise $v_{k}$ is given by:

$$R=\left[\begin{array}{cc} \sigma_{total}^{2} & 0\\ 0 & \sigma_{total}^{2} \end{array}\right],$$

where $\sigma_{total}^{2}$ is the total noise variance given in (6). Hence, for the given measured states $y_{k}$, the estimator predicts the next state by:

(13) $$\hat{x_{k+1}}=A\hat{x_{k}}+K\left(y_{k}-C\hat{x_{k}}\right),$$

where K is the gain of the estimator. The estimation error is calculated by:

(14) $$e_{k}=x_{k}-\hat{x_{k}}.$$

From (10) and (13) it can be shown that the estimation error has the following form:

(15) $$e_{k+1}=Fe_{k}+Bw_{k}+Ln_{k}-Kv_{k},$$

where

(16) F=(A−KC+LG).

Having presented both the environment and the target models, the next section presents the filter design to estimate the target position.

## 3. Filter Design

### 3.1. Minimax Filter

According to the analysis in the previous section, we consider that the intruder has an intelligent adversarial behavior and aims to maximise the estimation error as described in (15). As a result, the estimation error is increased in (11). Therefore, the filter design should prevent this from happening and should estimate the next step which minimises the error [20,21]. Thus, the minimax filter is modelled by a zero-sum game with two players. K is the playing policy of the first player and L is the playing policy of the second player [19,20,21,22,23]. Consequently, the estimation error is decomposed into two parts as follows:

(17) $$e_{k+1}^{K}=Fe_{k}^{K}+Bw_{k}-Kv_{k},$$

where $e_{k}^{K}$ is related to the process noise $w_{k}$ and the measurement noise $v_{k}$. To achieve the optimised filter gains, $e_{k}^{K}$ should be minimised [20,21].

The second part of the estimation error is given by [20,21]:

(18) $$e_{k+1}^{L}=Fe_{k}^{L}+Ln_{k},$$

where $e_{k}^{L}$ is related to the adversary disturbance noise $n_{k}$. To obtain the robust filter gain taking the worst adversary noise into consideration, $e_{k}^{L}$ should be maximised. Then, the game cost function is defined as follows [20,21]:

(19) $$J\left(K,L\right)=tr\left(\sum_{k=0}^{t_{h}}E\left[||e_{k}^{K}|{|}^{2}-||e_{k}^{L}|{|}^{2}\right]\right),$$

where $t_{h}$ is the time horizon. Conveniently, the cost function *J* is presented in the following form:

(20) $$J\left(K,L\right)=tr\left(\sum_{k=0}^{t_{h}}P_{k}\right),$$

where $P_{k+1}=FP_{k}F^{T}+BQB^{T}+KRK^{T}-LSL^{T}$ is the filter covariance matrix.

After defining the estimation error and the cost function, the goal of the designer is to find the game equilibrium by solving the zero-sum game to find the optimised filter gain $K^{*}$ that minimises *J* and the robust filter gain $L^{*}$ that maximises *J* in the case of worst adversary performance. This solution satisfies the following saddle point equilibrium of the zero-sum game:

(21) $$\left(K^{*},L\right)\leq \left(K,L\right)\leq \left(K,L^{*}\right).$$

It is trivial to prove that the game equilibrium solution can be deduced by:

(22) $$K=A\phi_{k}C^{T}R^{-1},$$

(23) $$L=A\phi_{k}G^{T}S^{-1},$$

given that

(24) $$\phi_{k}^{-1}=P_{k}^{-1}+C^{T}R^{-1}C-G^{T}S^{-1}G.$$

### 3.2. Kalman Filter

The Kalman filter is constructed by setting G=L=0. Therefore, the cost function can be represented by:

(25) $$J\left(K,L\right)=tr\left(\sum_{k=0}^{t_{h}}P_{k}\right),$$

where $P_{k+1}=FP_{k}F^{T}+BQB^{T}+KRK^{T}$. Then, the filter gain K that minimises the cost function J(K) is given by:

(26) $$K=AP_{k}C^{T}{\left(CP_{k}C^{T}+R\right)}^{-1}.$$

Both filters are iterative, starting with the initial information to estimate a new position. Then, the estimation error is calculated as the difference between the predicted state and the measured state. Next, the filter minimises the estimation error.

## 4. Results and Discussion

The proposed system was simulated in the MATLAB™ environment. The important simulation parameters are summarized in Table 1. The simulation was carried out for a room dimension of 10×10×3 m${}^{3}$ with a sampling period of 1 s and a target speed of 1 m.s${}^{-1}$. The transmitter, which was an array of LEDs working together as a single light source, was located in the centre position (0,0) of the room. The number of receivers was 25. The receivers were distributed on the floor with a separation distance of 2 m between every two receivers. It was assumed that the process noise covariance Q=0.01I and that the adversary added noise covariance S=0.09I, in line with [20,23]. The weighting matrix, which indicates the adversary behavior of the intruder, was given by G=0.025I. In VLC, the measurement noise covariance R is calculated using the parameters in Table 1. Due to the randomness of the target trajectory, the results were averaged over 1000 simulation run.

**Figure 2:**
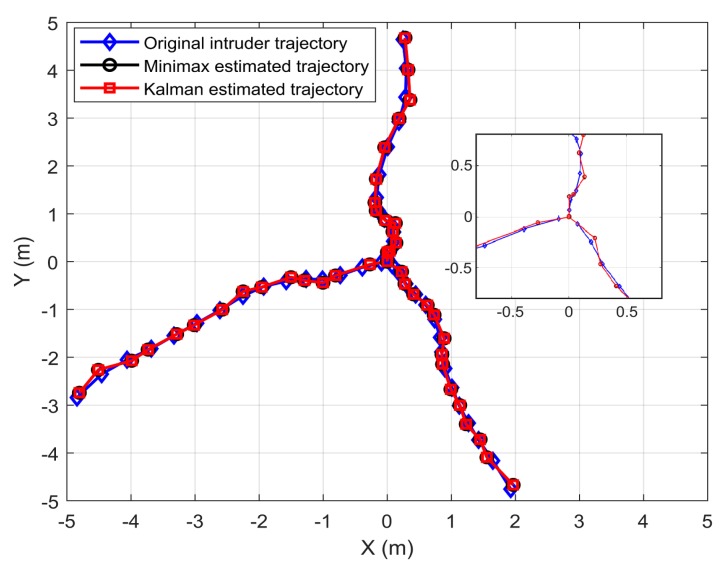
The actual and estimated (using minimax and Kalman filters) trajectories of the random paths of three different intruders, tracked using VLC.

Figure 2 demonstrates the actual and the estimated trajectories of three different intruder paths for the VLC system, using the minimax and Kalman filters. Even though the target trajectories were random, the motion of the targets were tracked with high accuracy by both filters.

**Figure 3:**
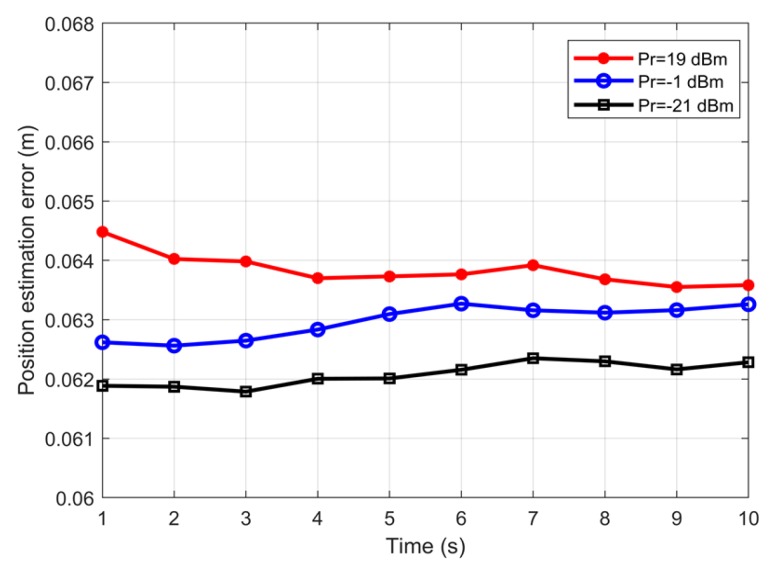
The estimated position error as a function of time for different power levels using a Kalman filter, assuming an ideal source.

**Figure 4:**
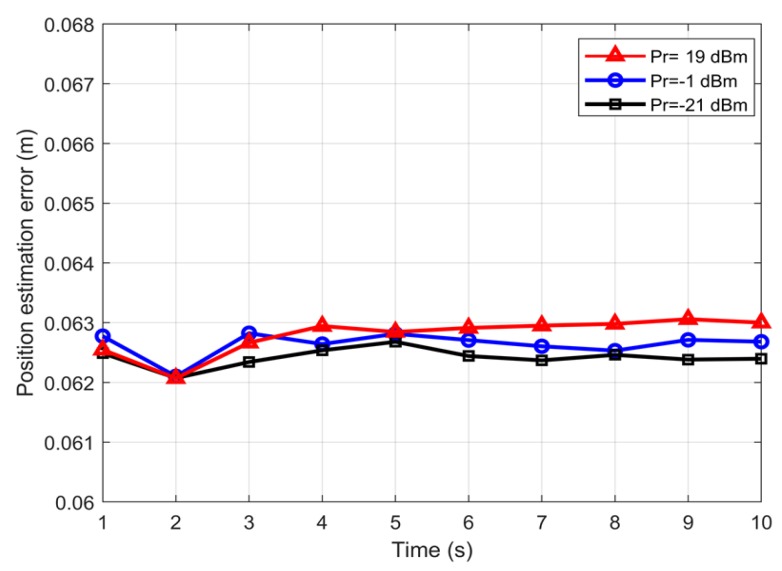
The estimated position error as a function of time for different power levels using a minimax filter, assuming an ideal source.

In order to analyse the position estimation error as a function of the received power, a hypothetical ideal source that provides uniform illumination at the receiver plan was considered. Note that with a Lambertian source, it is difficult to study the effect of received power on estimation error as the power varies throughout the room. In addition, this case shows the effect of the absence of the ambient noise on the performance of tracking. Figure 3 and Figure 4 show the estimated position error using the Kalman and minimax filters, respectively. Figure 3 clearly demonstrates that the estimation error in the Kalman filter was affected by the received power. In the stable regime, the lower received power offered a better estimation. The minimax filter approach had almost negligible variation in the estimated position error with the received power. The position estimation errors for the Kalman filters were ~0.064, 0.063 and 0.062 m for received powers of 19, −1 and −21 dBm, corresponding to the SNR values of 42, 55 and 57 dB. The minimax filter in Figure 4 had a position error of ~0.0625 m irrespective of the received power, clearly demonstrating the effectiveness and the advantage of minimax, especially when the received power is varying. The reduced negative impact of the received power is explained by the ability of the minimax filter to adjust the value ofthe playing policy of the second player, L, to compensate for higher values of R, modeling the noise due to the target evasive maneuvers and the environment, see (22) and (23).

**Figure 5:**
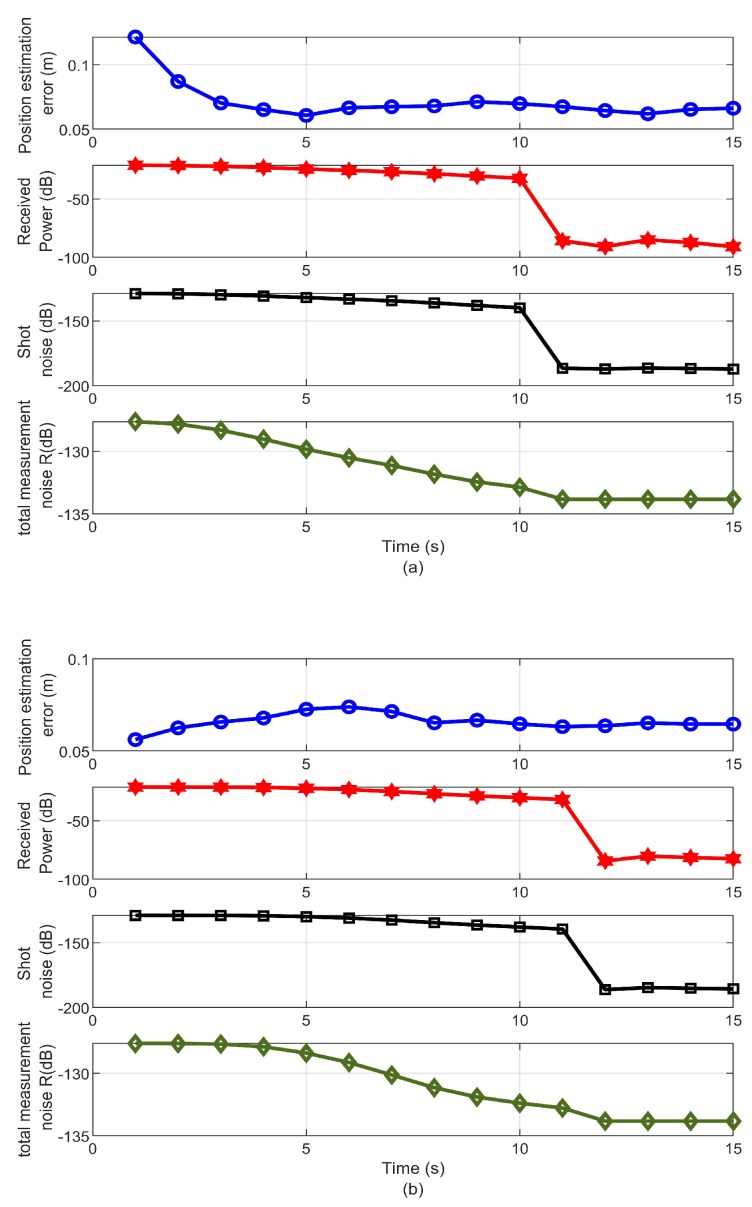
Relation between the estimation error, the received power, the shot noise and the total measurement noise using (**a**) Kalman filter and (**b**) minimax filter.

Figure 5 demonstrates the relation between the estimation error, the received power, the shot noise, and the total measurement noise using the minimax and Kalman filters. This figure demonstrates that for a shot noise dependent system, increasing the transmitter power had an adverse effect on the estimation using Kalman and minimax filters. A further explanation for the effect of the received power level on the accuracy of the estimation can be provided by referring to the value of total measurement noise *R*, which depends on the total noise covariance in (6). In this particular case, the thermal noise variance value was constant (−133.8 dB). However, according to (7) the value of the shot noise is linearly proportional to the received power. Therefore, increasing the received power increased the value of the shot noise. Consequently, it dominated the total noise covariance and hence the total measurement noise *R*, as shown in Table 2. This was limited by a lower bound of the received power value (−21 dB), where the thermal noise dominated the total measurement noise.

**Figure 6:**
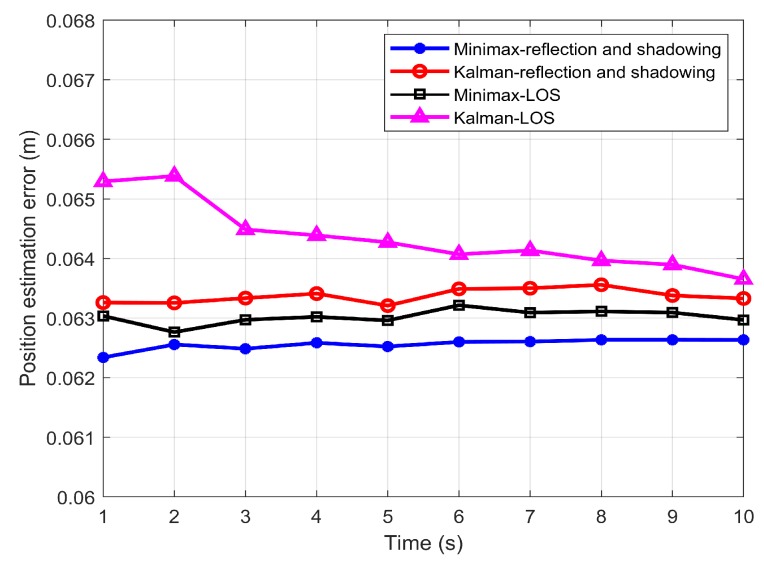
Position estimation error of the minimax verses the Kalman filters using line of sight (LOS) and shadowing.

Figure 6 presents the estimated position error as a function of time considering the LOS channel and channel with LOS, reflection and shadowing for the Lambertian source. Figure 6 illustrates that for both channel conditions, minimax offered a better estimation than the Kalman filter. In the system considering reflection and shadowing, the position estimation error was ~0.063 m and 0.062 m for the Kalman and minimax filters, respectively. Similarly, when the LOS component only was considered, the estimation errors had values of ~0.064 m and 0.063 m for the Kalman filter and minimax filter, respectively. This is because, in the case of the LOS, the received power was higher. Consequently, the shot noise given by (7) was comparable to the thermal noise given in (8). As a result, the total measurement noise R was increased, which reduced the estimation accuracy. By contrast, in the case of the realistic channel model, the reflection and shadowing reduced the received power. Therefore, the shot noise value Was negligible; hence, the dominant measurement noise was the thermal noise. Thus, the estimation performance was enhanced. The results were averaged over 1000 run, given rise to a standard deviation of the error was 0.0005106 for the Kalman filter and 0.00048146 for the minimax filter.

**Figure 7:**
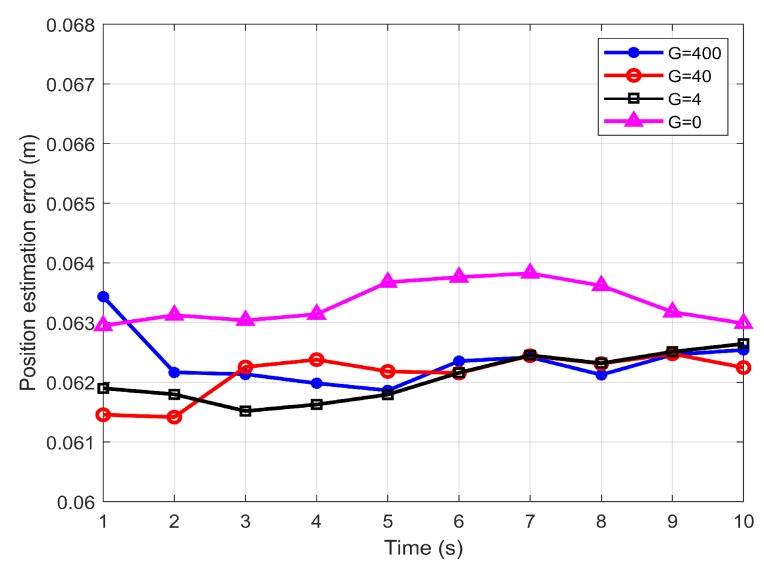
The influence of the adversary behavior of the intruder on position estimation.

Figure 7 shows the influence of the adversary behavior of the intruder, presented by the value of G. As depicted in the figure, the minimax filter achieved similar estimation error performance which stabilised around 0.0625 m regardless of the severity of the target’s adversary behavior. This result is justified by referring to the design parameter L in (23), which is adjusted according to the value of G. Therefore in the case G=0, which is equivalent to Kalman filter, the performance of the minimax filter was degraded.

**Figure 8:**
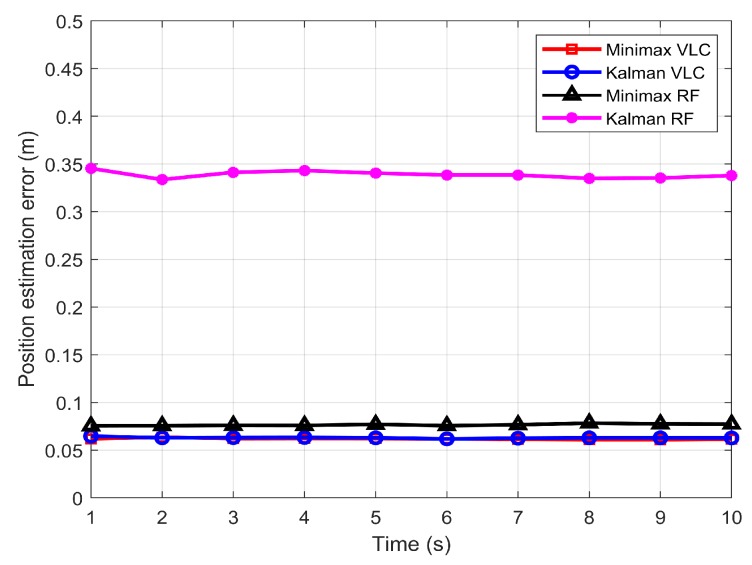
Comparison between the position estimation errors from minimax and Kalman filters using RF and VLC.

To understand the possible advantage of VLC intruder tracking in comparison to RF, RF-based intruder tracking was also carried out for similar scenarios. In the wireless sensor networks that use the 2.4 and 5.7 GHz industrial, scientific and medical (ISM) RF bands, the value of the measurement noise covariance R is 0.04 [19,23], which exceeds the VLC value by 12 orders of magnitude. Figure 8 presents the comparative performance of the minimax and Kalman filters in the RF and VLC environments. The figure clearly illustrates that the VLC offered better position estimation than the RF system. The performance difference between VLC and RF was higher for the Kalman filter than for the minimax filter. A performance difference of 16 cm is noticeable between RF and VLC for the Kalman filter, whereas the difference was 2 cm for the minimax filter, with the VLC system outperforming RF in both cases.

**Figure 9:**
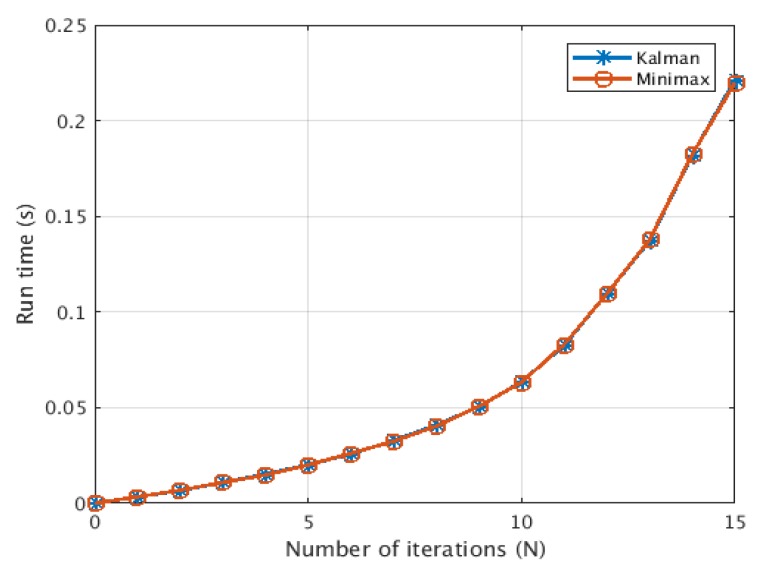
The computational complexity of the minimax filter algorithm.

In order to demonstrate the possibility of using this technique in real-time operation, we provide a brief analysis of the complexity of the minimax filter. We follow the analysis provided in [26] to analyse the computational complexity of the minimax filter algorithm. Table 3 shows that the minimax filter has a computational complexity of $O(N^{2.376})$ per iteration. This is comparable to Kalman filter computational complexity which also has the same complexity of $O(N^{2.376})$ [26,27]. Figure 9 shows that both minimax filter and Kalman filter had a comparable computational complexity performance with $O(N^{2.376})$ trend.

## 5. Conclusions

This paper has presented a comprehensive study of intruder tracking using VLC and minimax filters. A realistic simulation environment was developed including a LED source located in the ceiling employed as the VLC transmitter and PDs placed on the floor as receivers. The LOS, reflection and shadowing were considered. The results show that both minimax filter and Kalman filter tracked the intruder with high accuracy, with errors of less than 0.07 m and a comparable computational complexity performance of $O(N^{2.376})$. However, the performance of the minimax filter was slightly better than that of the Kalman filter and was less affected by the channel conditions and variation in power. The minimax filter also showed a stable estimation error performance of 0.0625 m, regardless of the severity of target adversary behavior. Minimax was also shown to significantly outperform the Kalman filter when RF was used in place of VLC.

## Figures and Tables

**Table 1:** Simulation parameters.

Symbol	Parameter	Values
$P_{R}$	Optical power by individual LED	20 mW
$n_{LED}\times \;n_{LED}$	Number of LEDs in the array	60×60
$\Phi_{c}$	Semi-angle	$60^{\circ }$
γ	Photodiode (PD) responsivity	0.54 A/W
$\Psi_{c}$	The receiver field of view	$60^{\circ }$
$A_{r}$	Receiver area	$1\times \;10^{-4}$ m${}^{2}$
$C_{pd}$	Capacitance of PD per unit area	1.12μFm${}^{-2}$
$I_{2}$	Noise bandwidth factor	0.562
$I_{3}$	Noise bandwidth factor	0.0868
$B_{w}$	Noise bandwidth	100 MHz
$G_{\nu }$	Open-loop voltage gain	10
Γ	FET channel noise factor	1.5
$g_{m}$	FET transconductance	30 mS
$I_{bg}$	Background current (silicon PD)	10 nA
ρ	Reflection coefficient	0.8

**Table 2:** The received power, the total noise covariance and the total measurement noise *R*, according to the simulation results.

Received Power (dBm)	Shot Variance $\sigma_{\mathrm{total}}^{2}$ (dB)	Measurement Noise R (dB)
19	−119	−119×I
−1	−139.4	−132.6×I
−21	−158.8	−133.8×I

**Table 3:** Computational complexity of the minimax filter.

Algorithm Line	Complexity
$x_{k+1}={\mathrm{Ax}}_{k}+{\mathrm{Bw}}_{k}+\Delta_{k}$	$O(N^{2})$
$P_{k+1}=FP_{k}F^{T}+BQB^{T}+KRK^{T}-LSL^{T}$	$O(N^{2.376})$
F=(A−KC+LG)	$O(N^{2.376})$
$\phi_{k}^{-1}=P_{k}^{-1}+C^{T}R^{-1}C-G^{T}S^{-1}G$	$O(N^{2})$
$K=A\phi_{k}C^{T}R^{-1}$	$O(N^{2.376})$
$L=A\phi_{k}G^{T}S^{-1}$	$O(N^{2.376})$
$\hat{x_{k+1}}=A\hat{x_{k}}+K\left(y_{k}-C\hat{x_{k}}\right)$	$O(N^{2})$
